# Social Validity of Pivotal Response Treatment for Young Autistic Children: Perspectives of Autistic Adults

**DOI:** 10.1007/s10803-022-05808-4

**Published:** 2022-11-24

**Authors:** Rachel K. Schuck, Patrick Dwyer, Kaitlynn M. P. Baiden, Zachary J. Williams, Mian Wang

**Affiliations:** 1 Gevirtz Graduate School of Education, University of California, Santa Barbara, Santa Barbara, CA 93106-9490 USA; 2 Department of Psychology, University of California, Davis, Davis, USA; 3 Center for Mind and Brain, University of California, Davis, Davis, USA; 4 Medical Scientist Training Program, Vanderbilt University School of Medicine, Nashville, USA; 5 Department of Hearing and Speech Sciences, Vanderbilt University Medical Center, Nashville, USA; 6 Vanderbilt Brain Institute, Vanderbilt University, Nashville, USA; 7 Frist Center for Autism and Innovation, Vanderbilt University, Nashville, USA

**Keywords:** Pivotal response treatment, Social validity, Naturalistic developmental behavioral intervention, Autistic perspectives

## Abstract

The social validity of autism behavioral intervention has been questioned. Naturalistic Developmental Behavioral Interventions (NDBIs) attempt to address some concerns, but it is unclear whether autistic people consider NDBIs socially valid. Social validity of an NDBI, Pivotal Response Treatment (PRT), was investigated through autistic adults commenting on videos of autistic children receiving PRT. Qualitative coding of responses generated three themes: respect for individuals; assessment of intervention implementation; and socioemotional considerations. Although video brevity limits the scope of the present study’s conclusions, participants highlighted PRT components that appeared socially valid (e.g., reinforcing attempts, following the child’s lead) and aspects appearing invalid (e.g., overemphasis on spoken language). Therefore, adjustments appear necessary for PRT to be fully acceptable to the autistic community.

## Introduction

Social validity is an integral part of interventions. Whatever the “objective” outcomes of the intervention, if stakeholders do not see the program as useful and acceptable, the intervention cannot be deemed successful (Schwartz & Baer, 1991; Wolf, 1978). The term *social validity* emerged from the discipline of applied behavior analysis (ABA)—a scientific approach to understanding and modifying behavior^1^—when it was realized that strict adherence to behavioral principles can ignore the feelings and opinions of intervention recipients (Kazdin, 1977; Wolf, 1978). Wolf (1978) argued that, in addition to evaluating behavioral outcomes, clinicians and researchers must also pay attention to stakeholders’ *perspectives on* and *feelings toward* intervention procedures, goals, and outcomes. Though social validity is currently seen as an integral part of intervention programs [e.g., both Horner et al. (2005) and Reichow et al. (2011) include it in their criteria for evaluating whether an intervention counts as “evidence-based”], the construct remains understudied (D’Agostino et al., 2019; Ferguson et al., 2018; Snodgrass et al., 2018).

Nonetheless, social validity is a broad concept with many different conceptualizations and methods of measurement. For example, some researchers stress the utility of “objective” measures such as allowing intervention recipients to choose between different intervention alternatives (e.g., Hanley, 2010) and “normative comparison” (e.g., Kazdin, 1977; Kazdin & Matson, 1981), where an individual’s behavior is compared to what is considered “typical” in order to identify “socially significant” intervention goals and outcomes. (It should be noted that normative comparison is often not in line with the neurodiversity approach, as it encourages normalization, and is not being endorsed here.) Others have emphasized the importance of more subjective measures, with researchers giving participants questionnaires (e.g., Berger et al., 2016; Luiselli et al., 2015; see Finn & Sladezcek, 2001 for a review) or conducting qualitative interviews (e.g., Leko, 2014). Researchers’ motivations for studying social validity also vary widely, from wanting to understand all potential effects of an intervention (such as opinions and outcomes not measured by traditional behavioral measures, e.g., adverse events), wanting to ensure program survival (i.e., if people do not like an intervention, it is likely to fail), and wanting to give intervention recipients a voice (Snodgrass et al., 2021). It is arguable that, from an ethics standpoint, the latter motivation is particularly salient. However, even when social validity and consumer satisfaction are assessed in research, the autistic perspective is rarely taken into account; instead, the focus is often on parents’ or interventionists’ opinions (D’Agostino et al., 2019; Hurley, 2012). While this may provide valuable information, as Snodgrass and colleagues put it, “claims of social validity require adherence to the aphorism, “nothing about us without us” (Charlton, 1998)” (Snodgrass et al., 2018, p. 170), and many researchers and advocates have pushed for more emphasis on the lived experience of autism (e.g., Depape & Lindsay, 2016; Tesfaye et al., 2019).

However, assessing social validity in young children can be difficult, especially when children do not reliably use spoken language. While researchers have begun to develop approaches to reach such individuals (e.g., Courchesne et al., 2021; Robinson, 2011; Tesfaye et al., 2019), autistic adults can also provide valuable insights on the acceptability of behavioral interventions. Indeed, while many autistic adults have been vocal regarding their negative intervention experiences (e.g., Cumming et al., 2020; see also Schuck et al., 2021), little formal research has investigated this, and many clinicians believe that such critiques mainly apply to more traditional forms of ABA (comments on a *Spectrum News* article about ABA highlight this debate; Devita-Raeburn, 2016). To bridge this divide, it is crucial researchers assess the social validity of both traditional and newer forms of ABA from autistic perspectives.

### Development of NDBIs and PRT

Many common interventions for young autistic children are based on behavioral principles. Discrete Trial Training (DTT; Smith, 2001), often viewed as “traditionally-practiced ABA,” is clinician-led and focuses on repetitive trials targeting discrete skills wherein a reinforcer (reward) is given after the child successfully responds to a demand. DTT gained popularity after Lovaas’ 1987 study, which proclaimed that intensive behavioral intervention significantly reduced autism “symptoms”; indeed, Lovaas claimed almost half of his sample was “indistinguishable from their normal [peers]” after intervention (Lovaas, 1987, p. 8). The apparent success of this study spawned myriad research studies and an entire ABA industry, though such dramatic results have not been replicated (Ospina et al., 2008; Sandbank et al., 2020).

However, traditional ABA models, such as DTT, are clinician-driven, with little emphasis on child motivation or interest. DTT has historically been conducted in contrived formats (e.g., at a table with flashcards) and often relies on rote memorization of targets and external reinforcement (e.g., snacks, stickers, etc.) for correct responding, leading to the hypothesis that this approach limits generalization of skills from clinical to real-life settings (Koegel & Koegel, 1995; Koegel et al., 1998). In an effort to improve upon these traditional models, researchers designed more naturalistic interventions focusing on generalization and increasing child motivation (e.g., Koegel et al., 1998; McGee et al., 1985). This class of interventions, termed Naturalistic Developmental Behavioral Interventions (NDBIs; Schreibman et al., 2015), combines behavioral principles with a developmental approach. Recent meta-analyses have found evidence for the effectiveness of NDBIs, particularly with regards to language, play, and social communication skills (Sandbank et al., 2020; Tiede & Walton, 2019), though such findings must be understood within the context of several methodological weaknesses (e.g., outcome boundedness and proximity; Crank et al., 2021).

Pivotal Response Treatment (PRT) is an NDBI model that seeks to target so-called “pivotal areas'' of a child’s development (e.g., motivation, responsivity to multiple cues, self-management, and social initiations) rather than isolated behaviors (Koegel et al., 2016). PRT emphasizes letting the child choose activities (*following the child’s lead*), creating *shared control of the activity* between child and adult, and presenting a combination of easy and difficult tasks relative to the child’s ability (*interspersing maintenance and acquisition tasks*). Moreover, it makes use of *natural reinforcement* in lieu of external reinforcement and calls for *reinforcing attempts*, where reinforcement is contingent upon child effort, without always expecting “correct” or “perfect” responses. PRT often utilizes a parent education model in which parents are taught PRT procedures and are encouraged to implement it with their child throughout the day. Several randomized controlled trials have found that parents are able to implement PRT with fidelity and that children exhibit improvements in expressive language after intervention (e.g., Gengoux et al., 2020; Vernon et al, 2019). Overall, PRT has been found to be a promising intervention in both single-case research (Bozkus-Genc & Yucesoy-Ozkan, 2016) and randomized controlled trials (Verschuur et al., 2014), though it should be noted that its evidence base faces similar methodological issues as NDBIs more broadly (Ona et al., 2020).

Those who study and implement NDBIs, and more specifically PRT, suggest they can provide enjoyable, beneficial learning experiences for autistic children (e.g., Vivanti & Zhong, 2020), and it has been argued that NDBIs such as PRT can theoretically be reformed to be neurodiversity-affirming (Schuck et al., 2021). However, even though NDBIs were developed with the child’s perspective in mind (e.g., child motivation, following the child’s lead), research on these interventions has rarely included social validity measures (e.g., Bozkus-Genc & Yucesoy-Ozkan, 2016; Callahan et al., 2017; D’Agostino et al., 2019). Even when stakeholder perspectives are included, teachers, clinicians, or parents are often asked for their opinions without considering the views of the autistic people receiving the intervention (D’Agostino et al., 2019; see Monahan et al., 2021 for an example of this in adult social skills interventions). Inclusion of the client perspective is particularly important, as many autistic individuals have been vocal about their criticisms of many behavioral interventions.

### Critiques of ABA

Emerging qualitative research supports claims that ABA practices can be experienced as traumatic by some recipients (Freitas, 2020; McGill & Robinson, 2020). Many critiques of ABA focus on three areas: use of aversive punishment, emphasis on compliance, and a focus on normalization. When these interventions were first developed, many used aversive stimuli, including electric shock and hitting (Lichstein & Schreibman, 1976; Simmons & Lovaas, 1969) to extinguish “negative” behaviors. Though these aversives have been mostly abolished, there are institutions still using them today (Neumeier & Brown, 2020). Additionally, it is important to note that aversive stimuli can extend beyond these extreme examples. Autistic people have reported that other intervention procedures (e.g., blocking engagement in preferred activities or providing exposure to uncomfortable sensory stimuli) can be perceived as extremely aversive to them (e.g., Bascom, 2015; Botha et al., 2021).

Similarly, some advocates and researchers (both autistic and non-autistic) feel that all behavioral interventions put too much emphasis on compliance, given their use of contingent reinforcement and physical prompting (I am a disillusioned BCBA: Autistics are right about ABA, 2020; Sandoval-Norton & Shkedy, 2019). They worry that emphasis on compliance will teach children that they lack autonomy, which could have dire consequences with regard to personal/physical safety, a particularly salient concern with regard to sexual consent, as autistic individuals are at higher risk of sexual victimization (Pecora et al., 2020).

Another issue highlighted by autistic advocates is ABA’s emphasis on normalization—that is, the apparent goal of interventions to make autistic people look “less autistic” (Dawson, 2004; Wilkenfeld & McCarthy, 2020), suggesting they may encourage camouflaging—or masking—of autistic behaviors (see Cook et al., 2021). Persistent pressure to suppress one’s true autistic self can be exhausting and harmful (Ne’eman, 2021); in fact, masking has been cross-sectionally associated with negative mental health outcomes, including suicidality (Cage & Troxell-Whitman, 2019; Cassidy et al., 2018; Hull et al., 2021), though more research is needed to establish causality and rule out various confounding factors (Williams, 2021). Unfortunately, it is hard to deny that the field has historically promoted neurotypical “normality,” as Lovaas’ (1987, p. 8) landmark paper identified participants being “indistinguishable from their normal friends” as the intervention’s ideal outcome. [As suggested by Bottema-Beutal et al. (2020), we use the term *neurotypical* to refer to individuals who fit society’s typical norms and who do not identify as having a neurological difference.] While not every interventionist today would take such a strong stance, many intervention goals and practices still align with neurotypical behaviors, for instance those around developing “social skills” (e.g., eye contact). Interview studies by Santhanam and Hewitt (2021) and Bottema-Beutel et al. (2016) suggest autistic individuals may feel that social skills interventions are ineffective and yearn for greater neurotypical understanding. Behavioral intervention also has a history of attempting to suppress autistic “repetitive and restricted behaviors and interests (RRBIs)” (e.g., Liu-Gitz & Banda, 2010; Ventola et al., 2016), such as hand flapping and vocal scripting, a practice that continues today. However, many on the spectrum do not see these behaviors as problematic; in fact, they may be used for self-regulation (Bascom, 2015; Charlton et al., 2021; Kapp et al., 2019), and negativity regarding such behaviors may stem more from stigma and neurotypical social norms than from intrinsic properties of RRBIs. Such efforts to suppress autistic behavior have led to calls for interventions to stop encouraging masking (Ne’eman, 2021; Roberts et al., 2020) and to some autistic advocates labelling ABA as “autistic conversion therapy” (e.g., Sequenzia, 2016), a parallel bolstered by Lovaas’ involvement in a study designed to reduce “feminine traits” in young boys (Rekers & Lovaas, 1974).

### Where Do We Stand Now?

These critiques of ABA certainly apply to traditional models, such as those espoused by Lovaas and colleagues. The question now is: are such critiques relevant to newer, naturalistic models? More specifically, how do autistic individuals feel about NDBIs? Do autistic people have concerns about these child-led, play-based, and seemingly strength-based interventions? When faced with this question, NDBI researchers and clinicians may feel NDBIs are respectful of the child, and that the critiques listed above only apply to less naturalistic approaches. Clinicians commonly refer to ways in which ABA has changed, arguing it is no longer abusive (again, see the *Spectrum News* article comments; Devita-Raeburn, 2016). However, a common internet hashtag, #YesAllABA (meaning, “Yes, all ABA is abuse”), clearly rejects this line of thinking. Furthermore, some researchers argue all interventions based on reinforcement of desired behavior, including NDBIs, must be abolished (e.g., Kohn, 2020; Mottron, 2017). Others have suggested that autism intervention may be conducted effectively and respectfully if (and only if) researchers and clinicians do more to take autistic individuals’ perspectives into account and base intervention goals on autistic—rather than neurotypical—behavior (Fletcher-Watson, 2018; Leadbitter et al., 2021; Schuck et al., 2021). With so many competing perspectives, it is necessary to elicit feedback directly from the most important stakeholders: autistic people themselves.

### Current Study

The current investigation aimed to begin exploring the social validity, from an autistic perspective, of a prominent NDBI model, Pivotal Response Treatment (Koegel et al., 2016), by eliciting feedback from autistic adults after they watched videos of children engaged in PRT sessions. Our primary research question was: How do autistic adults perceive Pivotal Response Treatment?

## Method

This study was approved by the University of California, Santa Barbara Institutional Review Board. Study advertisements were posted on social media (e.g., Facebook, Reddit) pages geared toward autistic adults and additionally sent to autism organizations. A variety of organizations were targeted, including those that were likely to have differing opinions on ABA (e.g., some groups were for autistic behavior analysts, while others espoused a specifically anti-ABA perspective). Almost 200 organizations were contacted; because not all organizations responded to the study team, we were unable to determine the exact organizations from which our participants came. All participants gave informed consent via a Qualtrics signature box before participating. Participants did not receive any compensation or incentive for completing the study survey.

### Procedure

The study was conducted via Qualtrics (Qualtrics, 2021). Individuals needed to be autistic (formally diagnosed or self-identified) and at least 18 years old to participate. After consenting, participants completed the Ritvo Autism Asperger Diagnostic Scale—Screen (RAADS-14; Eriksson et al., 2013). Though the RAADS-14 was not used as an inclusion criterion, scores were used to describe the sample overall. Next, participants were asked to watch five short video clips of children engaged in a PRT session and asked questions about the videos as well as demographic questions. The survey contained another section about participants’ views on intervention goals and practices; analyses of these questions will be presented elsewhere.

Participants were told that the purpose of the study was to learn about autistic individuals’ perspectives in order to hopefully improve interventions in the future. While they were told they would be watching videos of children engaged in a behavioral intervention, participants were not told that the specific intervention was PRT, ABA-based, or an NDBI, as we did not want to bias participants with this knowledge.

### PRT Videos

Participants were shown five short video clips (27–50* s*) of young (2–4 years old) autistic children engaged in a PRT session with an adult caregiver or clinician. Three male children appeared in the five videos; four contained child–parent dyads (including one child–mother dyad that appeared in two videos) and one contained a child–clinician dyad (this child was also shown in one of the child–parent videos). Two of the children were able to speak in short phrases; the other was not using spoken words to communicate. In each video, adults created opportunities for children to practice spoken language.

Four of the five videos showed adults implementing mostly PRT *maintenance tasks*, meaning responding to the adults’ prompts and/or questions was classified as an easy task for the child. In these cases, adults in the videos reinforced the child’s initial response by immediately giving them the natural reinforcer (i.e., they gave them the item or access to the activity they asked for). One video showed an adult targeting an *acquisition task* where the target behavior—approximating a word with more accurate pronunciation as opposed to making a more generalized utterance—was difficult for the child, who was not yet using spoken language or imitating sounds. When the child continued to only respond with his generalized utterance, the adult therefore contingently withheld reinforcement, in line with PRT/NDBI guidelines for teaching first words (Jobin & Schreibman, 2020; Koegel et al., 2003). This video was included to ensure participants saw representative videos of PRT, including what happens when children do not respond with the target behavior after adult prompting.

All video clips were chosen because of the following reasons: (1) parents in the videos were judged by the first and third authors to meet fidelity of implementation of PRT using the criteria laid out in Bryson et al. (2007) (i.e., using all PRT components correctly), (2) they were all determined to be an accurate representation of a typical PRT session based on the first and third authors’ experiences implementing PRT, (3) they collectively represented both the target of maintenance and acquisition tasks, and (4) they include children with varying spoken language levels to illustrate how PRT can look with different children. See Table 1 for definitions and video examples of each of the PRT components.

**Table 1:** Explanation of PRT Components & Examples from Videos

PRT component	Definition	Example from Video Clips
Learning opportunities	The adult creates an opportunity for the child to communicate by gaining control of an item or part of an activity that the child wants to access; may be deliberately constructed or occur incidentally	Video 1: The adult stops spinning the child and waits for him to request it again Video 2: When the child reaches toward the kitchen counter, the adult says “up?” and waits for the child to say “up” Video 3: The adult says “what do you want?” and the child says “I want box please.” Video 4: The adult says “go fast or go slow?” and the child says “go fast” Video 5: The adult says “zero?” and the child says “zero.”
Child Choice/following the child’s lead	The child is allowed to pick the activity. The adult does not force the child to pick a specific activity or keep them engaged in an activity that they no longer want to participate in	Video 3: The child puts down the jack in the box and grabs a star toy. The adult moves on and begins engaging with the child with the newly chosen toy Video 5: The adult continues a number activity when the child is laughing and visibly engaged in it
Shared control	The adult and the child share control of the activity. The child can engage freely with the items/part of the activity that they have control over. The adult maintains some pieces/part of the activity so they can encourage the child to communicate	Video 3: After the child picks the star toy, the adult takes some of the star pieces to create learning opportunities
Interspersing maintenance and acquisition tasks	The adult targets a combination of easy and difficult tasks relative to the child’s ability	Video 2: The adult will sometimes accept the child’s generalized response of “ooh”, but sometimes pushes for an approximation closer to the word “up.” • Maintenance: Adult says “pan?” and gives the child the pan when he says “ooh” • Acquisition: Adult says, “up?” and waits to reinforce until child says something approximating “up” (e.g., “uhh”) Video 4: The adult creates opportunities where the child responds to model prompts (which is easier for the child) and opportunities where the child is expected to generate their own words (which is a bit harder for the child) • Maintenance: Adult says “crash door?” and reinforces the child when he says “crash door” • Acquisition: Child says “go.” Instead of reinforcing right away, the adult asks, “Go where?” and reinforces once the child says “go up.”
Natural reinforcement	The adult reinforces the child’s requests with whatever reinforcer would normally occur after one makes such an utterance (usually access to more of the activity or item that they requested). The reinforcement is directly tied to the child’s utterance	Video 1: The adult spins the child when the child says “circle mommy” Video 3: The adult hands the child the orange star when he says “orange star” Video 4: The adult says “go fast or go slow?”, the child says “go fast” and the adult pushes the child on a scooter quickly
Contingent reinforcement	The adult reinforces the child with more access to an activity or item contingent on them requesting it. The adult does not reinforce the child if they do not request it	Video 1: The adult spins the child around as soon as he says “circle mommy”. (Providing contingent reinforcement for a “correct” response) Video 2: The adult does not lift the child up when he says “ooo” instead of a more clear approximation of the word “up”. (Remaining contingent for an “incorrect” response)
Reinforcing attempts	The adult provides reinforcement contingent upon child effort, without always expecting “correct” or “perfect” responses	Video 4: Adult says “crash door?”, the child says “craa door” and the adult pushes his scooter until it bumps the door (i.e., providing reinforcement) Video 5: The adult says “seven?”, the child says “ss” and the adult playfully puts the number seven puzzle piece in the puzzle (i.e., providing reinforcement)

See Bryson et al. (2007) and Koegel et al. (2016) for more details regarding components of PRT implementation

### Survey Questions

Three questions were presented after each video. Participants were first asked the degree to which they agreed with the following statements using a 6-point Likert scale (1 = strongly disagree, 2 = disagree, 3 = slightly disagree, 4 = slightly agree, 5 = agree, 6 = strongly agree): (1) *I find this intervention acceptable*; (2) *I would suggest the use of this intervention for other autistic children*. Next, participants were provided with a textbox and asked, *What do you think about the intervention in the video*? Participants were thus presented with a total of 10 Likert scale questions (two per video) and five open-ended questions (one per video).

### Participants

Participant names were reviewed before data analysis to ensure no duplicates were included. Review of participant names, along with the fact that this study was unfunded and provided no incentive for participating, likely limited any fraudulent activity that can be common with online survey studies (Teitcher et al., 2015).

A total of 192 autistic individuals (81 female, 29 male, 39 nonbinary/genderqueer, 43 other/unknown; M_age_ = 34.67, SD_age_ = 11.02) answered at least one of the survey questions. Most (n = 105) lived in the United States (46 lived elsewhere; 41 did not specify). Of the 162 who answered the question about race/ethnicity, a large majority identified as White (n = 143). Forty-eight participants (25.0%) reported receiving behavioral intervention as a child, 80 (41.7%) reported not receiving it, and 33 (17.2%) were unsure (31 [16.1%] did not answer). Most (n = 140 [72.9%]) reported having a clinical diagnosis of autism, with an average age of diagnosis of 25.04 years (SD = 13.11; range 2–62). Of the 131 participants who answered the question about age of clinical diagnosis, 37 (28.2%) received a diagnosis before age 18. The remaining 52 participants (27.1%) reported not having an official diagnosis but self-identified as autistic. RAADS-14 scores ranged from 6 to 42 (M = 31.99, SD = 6.97); only four participants (2.1%) scored below the autism cutoff of 14, all of whom reported a formal diagnosis.

Of these 192 participants, 175 (91.1%) responded to at least one of the five open-ended questions and 170 (88.5%) answered at least one question in response to all five videos (see Table 2 for the number of participants who responded to each question). Participants who stopped the survey before watching and providing feedback on all five videos were not significantly different from those who watched all videos in terms of diagnostic status (clinical diagnosis versus self-identification, *χ*^2^ = 2.41, *p* = 0.121) or age (β = 0.028, *p* = 0.214). As other demographics questions (e.g., gender, ethnicity, behavioral intervention as a child) were presented at the end of the survey, it is not possible to analyze differences in attrition based on these traits.

**Table 2:** Number of participants who answered each question

	Intervention is acceptable	Suggest this intervention for others	What do you think about the intervention?
Video 1	189	185	169
Video 2	180	180	162
Video 3	176	175	153
Video 4	170	169	149
Video 5	168	167	144

In the first two questions, participants were asked to rate the degree to which they agreed with the statements using a 6-point Likert scale. The third question was open-ended; participants could enter their response in a text box

### Data Analysis

Descriptive statistics regarding the two Likert questions are presented. An overall *acceptability* and *suggestion* score was calculated for each participant by taking the average of their responses to each question across videos. Descriptive statistics for these overall scores are also presented. Independent-samples t-tests were conducted to assess whether participants’ average *acceptability* and *suggestion* scores were related to whether they reported receiving behavioral intervention as a child or not (those who indicated they were unsure were not included in this analysis).

Participants’ responses to the open-ended question were analyzed following the six iterative steps of reflexive thematic analysis laid out in Braun and Clarke (2006, 2019): data familiarization, data coding, generation of initial themes, developing and reviewing themes, refining themes, and writing the report. Coding was done via *Dedoose* software (SocioCultural Research Consultants, 2021) and utilized a combination of inductive and deductive coding (Miles et al., 2018). To begin analysis, the first author (RKS) read a subset of participant responses and developed an initial set of inductive codes. These were combined with deductive codes based on PRT (Bryson et al., 2007) and NDBI (Frost et al., 2020) fidelity as well as literature on autistic adults’ views of ABA (e.g., Cumming et al., 2020; Michael, 2018; Sequenzia, 2016; Sparrow, 2016). The second, third, and fourth authors (PD, KMPB, ZJW) all reviewed subsets of the data using the initial codes and made modifications. To enhance the credibility and trustworthiness of the analysis, three authors (RKS, PD, KMPB) then used the codebook to independently code subsets of responses. The three authors met multiple times to discuss disagreements and refine code definitions and codebook structure. After coding responses from approximately 70 participants, all three authors felt no more codes needed to be added or changed. RS then coded responses from all 175 participants; PD reviewed all code applications, and any disagreements were reviewed and discussed amongst the three coders until consensus was reached. Code applications were then sorted into overarching themes (while we use the word “theme” for clarity, some of our “themes” are more like topic domains, in that they summarize participants’ responses about a particular topic, e.g., “reinforcement”; see Braun & Clark, 2020 for a discussion of themes versus topics). Though we recognize that quantifying qualitative data is sometimes controversial (Braun & Clark, 2020), we opted to also include the number of participants who endorsed each theme (see Table 3), as some themes were much more common than others, though we felt all were important to present.

**Table 3:** Subtheme endorsement frequency

Theme & subtheme	Frequency (N (%))
Respect for individuals	
Overemphasis on language	54 (30.8%)
Individualization of intervention	85 (48.6%)
Interpretation of child’s behavior	63 (36.0%)
Assessment of intervention implementation	
Following the child’s lead	98 (56.0%)
*Not* following the child’s lead	70 (40.0%)
Contingent reinforcement	
Favorable	13 (7.43%)
Not favorable	22 (12.6%)
Reinforcing attempts	18 (10.3%)
Not reinforcing attempts	59 (33.7%)
Amount of engagement	
Just right	49 (28.0%)
Too much	66 (37.7%)
Too little	24 (13.7%)
Where’s the intervention?	32 (18.3%)
Socioemotional considerations	
Child affect	
Positive	108 (61.7%)
Negative	66 (37.7%)
Parent–child bonding	
Positive	14 (8.0%)
Negative	5 (2.9%)
Intervention leads to harm	27 (15.4%)

All percentages are out of 175, as this was the number of participants who answered at least one open-ended question

#### Author Positionality

Team coding by consensus allowed the authors to take a reflexive approach to analysis where their identities and experiences were seen as analytic assets as opposed to barriers to ‘objective’ truth. Our backgrounds are thus integral to our understanding of our qualitative data, as all data was coded and discussed by both autistic and non-autistic authors with and without clinical experience. Those with behavioral intervention experience likely viewed our qualitative data through a PRT-lens; that is, they probably saw PRT principles at play in participants’ responses even if they were not explicitly referenced. On the other hand, the autistic authors, who had little or no behavioral intervention background, were more immersed in ideas widely discussed in autistic adult communities, such as the Double Empathy Problem (Milton, 2012), and likely interpreted responses accordingly.

The first four authors are graduate students. Two (RKS and KMPB) identify as non-autistic, both of whom are enrolled in an education doctoral program and are certified in implementing PRT. While the first author’s (RKS) emphasis is on research, particularly regarding intervention social validity, the other’s (KMPB) focus is on providing clinical services, as she is a board certified behavior analyst at a community agency. The other two student authors (PD and ZJW) both identify as autistic. Neither autistic author has personal experience being a client/recipient of ABA-based intervention, and neither autistic authors’ careers involve delivery of ABA-based intervention, although ZJW previously received training in PRT implementation through the University of California, Santa Barbara. The second author (PD) is a doctoral candidate in developmental psychology whose primary research interest is sensory processing and attention in autism, although he also studies autism community views regarding neurodiversity and intervention. The fourth author (ZJW) is an MD/PhD candidate and psychiatrist-in-training whose research focuses primarily on autistic adults and the various individual differences that predict co-occurring physical and mental health problems in this population. The remaining author (MW) is a non-autistic professor of education, focusing on inclusive education and implementation of evidence-based practices for children with disabilities. It should be noted that all of the authors approached this study from the perspective of acknowledging issues (historical and current) with behavioral intervention—including PRT—and wanting to improve intervention without abolishing it. This viewpoint is expressed in the authors’ prior writing on this subject (Schuck et al., 2021).

## Findings

### Quantitative Questions

Average ratings of acceptability of the intervention in each video ranged from 3.09 (SD = 1.62) to 4.72 (SD = 1.36). Average ratings of suggesting the intervention be used with other children ranged from 2.87 (SD = 1.54) to 4.49 (SD = 1.40). The lowest average ratings for both questions were for the video depicting the acquisition tasks (i.e., the adult holding out for a clearer approximation of a word; Video 2 in Table 1). The mean and median overall *acceptability* scores were, respectively, 4.11 (SD = 1.14) and 4.33. The mean and median overall *suggestion* scores were 3.74 (SD = 1.16) and 4.00.

While those who indicated they had had behavioral intervention had slightly higher mean *acceptability* (4.29 vs. 4.00 out of 6) and *suggestion* (3.98 vs. 3.64 out of 6) scores than those who indicated they had not, the differences were not statistically different (*acceptability: t*(125) = 1.53, *p* = 0.128; *suggestion: t*(125) = 1.65, *p* = 0.101).

### Open-Ended Responses

Three themes were generated based on comments from the 175 participants who answered at least one open-ended question: Respect for Individuals; Assessment of Intervention Implementation; and Socioemotional Considerations. Each theme is further broken up into subthemes. Frequencies of participants mentioning each theme can be found in Table 3.

#### Respect for Individuals

The theme of respect for individuals was broken into three subthemes: overemphasis on spoken language, individualization of intervention, and interpretation of child’s behavior.

##### Overemphasis on Spoken Language

Many participants felt that the intervention’s emphasis on improving spoken language skills was misplaced: that spoken language should not be prioritized over other means of communication that might be more accessible for some children. For example, one participant commented: “I don't believe in forcing non-verbal autistic children to speak or learn how to pronounce words properly. There are other ways to teach an autistic child to communicate, like ASL or using an electronic aid." Similarly, another said: “Spoken language is important and working towards it is fine–wonderful even–but are we also offering AAC? Sign language? PECS? How else are we giving this child a voice? Not all Autistic people can speak or prefer spoken language/find it easy in all situations.” These participants felt *any* communication should have been honored by the adults in the videos and that other communicative techniques (e.g., gestures, alternative and augmentative communication devices, American Sign Language, etc.) should have been taught along with or instead of spoken language. Many participants felt the emphasis on spoken language was not only misguided, but also disrespectful: “It was dismissive and manipulative to insist on only verbal communication" said one participant. Even when children were saying intelligible words, some participants wondered whether the adults would have honored children’s requests if they had used a non-verbal gesture. For the sake of identifying disconfirming evidence, it should be noted that three participants felt emphasizing language was acceptable when the child seemed able and willing to speak (e.g., “In this case, it seems like the child is ok with speech”), and another two clearly stated they felt language was not being prioritized.

##### Individualization of Intervention

A large proportion of participants (n = 85) highlighted that intervention activities would need to be tailored to each individual child. Many noted that the children in the videos seemed to enjoy the activities in which they were engaged (e.g., spinning around in a circle, riding a scooter, playing a game in which an adult humorously shouts), but that such enjoyment is likely not generalizable for all autistic children. For example, one participant stated: “I expect this specific intervention to be good for some and bad for others, so consent is really important." Especially salient were participants’ concerns about sensory sensitivities, such as not liking to be touched, being averse to excessive vestibular stimulation, and sensitivity to loud noises (e.g., "I was a little worried about the boy's reaction to the sudden loud speech from the adult, but he seemed fine with it. Others may not be;” “Bottom line for me is that the child seems to enjoy [being swung in a circle]. I think many autistic kids would be distressed by that but I think it's okay for this child”).

##### Interpretation of Child’s Behavior

Almost a third of participants mentioned some kind of hypothesis about what the child in the video must have been thinking or feeling (for example, “The little boy knows that the adult knows exactly what he (the boy) is asking for … Insisting on things like this cause stress for the child and adult alike;” “Her voice appears to be bothering his ears and he might be sensitive to noise. It is clear to me he is bothered by all the noises from the caretaker and the toys. He is slipping down in her arms trying to get away from her voice”). In other words, these participants were actively trying to understand and empathize with the children’s experiences. It does not seem unreasonable to infer that these participants considered children’s mental states and experiences to be an important consideration in determining whether interventions were acceptable, and by extension, that the participants would expect adults delivering intervention to likewise strive to understand how children experience intervention. For example, the comment, “The child seems uncomfortable and like they’re trying to acquiesce as much as possible to prevent the adult from getting more insistent and forceful” suggests that, instead of becoming more demanding, the adult should recognize how the child is feeling and change what they are doing.

#### Assessment of Intervention Implementation

Participant responses to the videos included reference to the following PRT fidelity components: Following the Child’s Lead, Contingent Reinforcement, and Reinforcing Attempts. Two additional subthemes related to intervention implementation were Amount of Engagement and “Where’s the Intervention?”.

##### Following the Child’s Lead

Over half of the participants highlighted positive instances of the adults in the videos following along with what the child was interested in doing (e.g., “It looks like the caretaker quickly adjusts when the child expresses disinterest in the learning game & I think that is good”) and honoring the child’s wishes when they wanted to do something else (e.g., “I'm glad the therapist asked if the child wanted to work on the Alphabet and respected when the child indicated that they didn't want to”). This suggests that following the child’s lead is a highly acceptable component of PRT. A subset of 25 participants suggested or explicitly mentioned that the adults received *consent* from the child to participate in the activity. For example, one participant stated, “The child is giving consent and is requesting it. That's great. If another child was also able to give consent and enjoyed it then that would also be great.”

However, a large proportion of participants (n = 70; 40%) also identified areas where it seemed the adult was *not* adequately following the child’s lead, either by interrupting their play too often or guiding them to do something the adult wanted to do. One participant connected this lack of child choice to autistic masking: “The woman would not let the child play the way he wants to, even though his way of playing was completely harmless. Autistic play styles aren’t somehow worse and don’t need to be corrected.” Many participants were concerned about how some adults obtained control of the activities in order to create opportunities for the child to speak (termed *shared control* in PRT). For example, some felt adults had too much control over the activities and that activities (e.g., scooter riding or spinning in circles) were being interrupted unnecessarily in order for the adult to promote spoken language. For example, one participant commented, “When [the child] tries to pick a toy, she takes it from him and forces him to talk to get what he was already getting for himself and then forces him to play with it how she wants to play, not how he does.” A similar sentiment is echoed in another comment: “It seems like she continuously kept stopping playing with him even though he was obviously having fun and wanted to continue to force him to speak, which seems manipulative.”

##### Contingent Reinforcement

Eleven participants brought up the reinforcement seen in the videos in a positive or neutral light. Multiple comments praised how the reinforcement in one of the videos was the adult’s humorous way of speaking, as opposed to the delivery of an object or activity (e.g., “She reinforces his participation by exaggerated movements that he clearly finds humorous;” “It was cute to see that the kid seemed to be mostly rewarded by the adult's overtly joyful reaction”). Others pointed out that natural, as opposed to external, reinforcement was being used, and that this could be a good thing (e.g., “The therapy is honoring the child's voice/agency and showing the child that when they ask they get the desired activity;” “The child is being asked to make a choice, and that choice is then acknowledged with follow-through;” “Demonstrating how a child can ask for something and then encouraging them to ask, when the thing they are asking for is inherently rewarding to the child…is a good way of fostering back and forth communication”).

However, 19 participants responded negatively to the use of any reinforcement. Many of these participants felt that children should not be made to “perform” in order to access enjoyable activities (e.g., “Again, she's requiring him to perform. She probably thinks she's "letting him play" but she's really not, she's giving him a small reinforcer to continue”) and that fun activities should be readily available regardless of child engagement in the desired behavior (e.g., “Fun should be for fun, not for rewards. Kids need to have unconditional relationships with trusted adults and she is imposing conditions;” “I think pressuring Autistics to say what NT [neurotypical] adults deem “right” and using mom’s play and affection as a reinforcer is fundamentally wrong;” “The child has to do a lot of oral speech to get teeny tiny flashes of fun out of the adult.”). These participants seemed to feel that, regardless of whether an activity was “fun” or not, reducing adult–child interactions to antecedent-behavior-consequence contingencies was unfair and could damage relationships.

##### Reinforcing Attempts

Eighteen participants commented that they appreciated how adults reinforced the children’s verbal attempts, even when they were imperfect (e.g., “They are not discouraged for a "wrong answer" and the person proceeds without expecting the "right" answer;” “I am glad the therapist is allowing the child to answer to the best of their ability; the word isn't flawless but the child is engaging and trying;” “Lays no demands on the child to "speak correctly" in order to "play on", and does not take anything away when failing to get the "correct" response.” All positive comments regarding reinforcing attempts were in response to two videos in which the children were highly engaged in the activity and adults were targeting maintenance tasks (Videos 4 and 5 in Table 1).

Fifty-nine participants noted instances where attempts were *not* being reinforced, mostly in response to the video in which the adult attempted to push a non-speaking child to approximate a word more closely (Video 2). As this was identified as an acquisition task for the child, the adult contingently withheld reinforcement and did not reinforce the attempt. Some participants worried that being dismissive of a child’s communicative attempts might convey to the child that their communication was not satisfactory and could ultimately affect their self-esteem. Other participants also felt it was unfair to push a child to say something that was obviously not currently in their repertoire, and that this could lead to frustration (e.g., “if it is clear to me what the child is communicating, it likely is clear to the parent. This makes things very frustrating for children”), lack of trust between child and adult (e.g., “The child might not be able to say the word yet, so why deny? It feels like teasing, bullying;” “This could also harm their relationship with their parent as they learn they can't trust their parent to listen and care for them.”), and even long-term harm (“Kid is clearly communicating in the way he knows how, adult clearly understands him but is refusing to acknowledge that communication and forcing a specific "right" way to ask. Bad for learning, emotional well-being, and communication skills in general.”). Similar to the last quote, another participant mentioned that this could be detrimental specifically with regards to communication skills (“The child is now no longer engaged in attempting to communicate their needs and wants. The communication process was halted because it wasn't perfect.”), which is the opposite of what the adult was trying to do.

##### Amount of Engagement

Comments regarding the engagement of the child and adult in the videos were made by 102 participants and fell into three categories: just right, too much, and too little. Many comments regarding “just right” engagement highlighted that the children looked highly engaged in activities and that adults were positively supporting such engagement by allowing the children space to explore and play and not forcing certain behaviors such as eye contact (e.g., “I like that the child is not being required to make eye contact or to sit up straight;” “I liked that the adult was not insisting on the child being there beside them, but rather interacted with the child as they were doing whatever they were doing;” “The child has freedom of movement, is enjoying a snack, and is being gently encouraged but not pressured by the adult”). Comments regarding “too much” engagement touched upon the ways in which the adults were “overbearing” (a word specifically used by four participants) towards the children (e.g., “Let the child play in peace! The adult is trying to take over everything and constantly talking;” “Too much rapid fire talking… even watching I get like I couldn’t breathe because the adult was talking too much and left no room for me to think;” “She isn't letting him play on his own, she's far too overbearing in getting him to play with things she suggested and he also doesn't look that thrilled with the task. He is clearly trying to get away from her and she keeps putting her hands in his face.”). A smaller subset of participants pointed out the lack of engagement sometimes displayed by the children, for instance by highlighting how children looked disinterested or bored (e.g., “This kid is clearly not engaged and wants to be doing something else.”).

##### Where’s the Intervention?

Some participants (n = 32; 18.3%) indicated that they were unsure of what the intervention was, as it appeared that they were just watching videos of adults and children playing together in a usual, non-therapeutic manner. One participant stated, “I wouldn't necessarily call it an intervention as it clearly looks like a parent spending time playing with their child.” Another similarly said, “I’m not recognizing any ‘intervention’ – just playing together.” Other participants indicated that what they saw in the video seemed like “just parenting” and that the adult in the video did the “same as a parent would do for a neurotypical child who had attained a similar degree of language ability.” These participants seemed to think that the PRT implemented in at least some of the videos looked just like everyday life.

#### Socioemotional Considerations

Participant responses regarding socioemotional considerations fell into three subthemes: Child Affect, Parent–Child Bonding, and Intervention Causes Harm.

##### Child Affect

Many participants commented on the affect of the children in the videos, with over half of the participants (n = 108; 61.7%) mentioning instances where a child displayed positive affect. Participants remarked that children were “having fun,” “enjoying” the activity, and “happy.” Some participants connected positive affect to effective learning (e.g., “The child is clearly really enjoying this method, and it is encouraging them to use their vocabulary in a tactile manner;” “It looks like the kid is enjoying it a lot, so it's probably super effective.”). On the other hand, a substantial number of participants (n = 66; 37.7%) also remarked that children were displaying negative affect, with many connecting this negative affect to aspects of the videos that were related to PRT intervention. For example, one participant explained how the adult getting shared control of a toy led the child to stop enjoying it: “Look at the drop of the child's mouth when the adult puts their hand on top of [the toy]…It really seems like they stopped enjoying the toy as much.” Others highlighted how pushing too much during acquisition tasks can cause distress: “The adult excessively trying to get the child to say “up” is clearly causing the child frustration.”

##### Parent–Child Bonding

A small proportion of participants (n = 19; 10.9%) mentioned the relationship between the adult and child in the video. Most (n = 14) felt that the interactions in the videos provided opportunities for building strong bonds and developing trusting connections (e.g., “The adult and the child seemed to have built a relationship where the child trusts the adult. They have mutual respect.” “The little boy looks like he's having a great time, and his mom is being loving, safe, and gentle. They look like they're bonding.”). However, a few participants (n = 5) did comment that the intervention might damage such relationships. One participant, an autistic parent of an autistic child, stated, “I can say this kind of interaction does not build relational connections.” Another participant said, “The child doesn't look interested, happy, or that he's spending time with the woman by choice; I don't see any meaningful emotional tie between them, either.”

##### Intervention Causes Harm

Some participants (n = 27; 15.4%) mentioned that the interventions shown in the videos could cause the child harm. Most of these comments alluded to harm related to the child’s well-being. One participant explained how they thought behavioral intervention might be related to masking and its impact on mental health:

Autism is not a bunch of “behaviors,” it is a different way of processing sensory information, and so Pavlovian behavior conditioning does not help the autistic person in question, it only makes the autistic person more “acceptable” to others, often at the expense of mental well-being.

Similarly, another participant worried that exposing children to too many behavioral learning opportunities would “steal their childhoods, their relaxation time, and their sense of self-worth.” Others felt that this type of intervention could lead to children thinking they always need to comply with adults: “This is why 'withholding' (e.g. a snack, toy, or activity) until a child complies is so dangerous- it teaches the child that their wants and needs are less important than pleasing the people around them.” One participant highlighted how this could cause problems down the road: “She is touching him constantly, which is probably overwhelming for him and is teaching him to override boundaries, which puts him at risk of future harm.”

## Discussion

Autism intervention programs have historically incorporated little input from autistic individuals. While NDBIs such as PRT were partially designed to be an improved version of previous behavioral intervention models due to their play-based nature, emphasis on child motivation, and absence of punishment (Vivanti & Zhong, 2020), critiques of ABA practices from within the autism community remain (see Des Roches Rosa, 2020 for a critique of PRT specifically). This study intended to begin exploring the social validity of PRT from an autistic perspective by eliciting feedback about the intervention directly from autistic adults by having them watch and give feedback on five short video clips of children engaged in a PRT session. While participants’ quantitative ratings of the video clips varied both by video and by participant, acceptability scores suggested that participants overall “slightly agreed to agreed” that the intervention they saw was acceptable. Overall suggestion scores were somewhat lower, with the average overall score falling in the “slightly disagree to slightly agree” range, perhaps reflecting that what is acceptable for one child may not be acceptable for another. These findings should, on the one hand, be fairly encouraging, since aspects of PRT depicted in the videos are overall seen as somewhat acceptable by members of the autistic community. However, on the other hand, this should also be somewhat alarming, as the variation in scores suggests there are contexts in which PRT is not seen as acceptable. These preliminary results should nonetheless be interpreted with caution, given that the two Likert scale questions have not been subjected to psychometric validation. As such, it will be impossible to know how individuals interpret and choose answer choices (e.g., what makes someone choose “slightly disagree” versus “slightly agree”?) until validation work, such as cognitive interviewing, is conducted.

Though these limitations limit our ability to interpret our quantitative findings, participants’ written responses after each video helped elucidate why some video clips were seen as more or less acceptable than others. Thematic analysis of these qualitative responses generated three themes: Respect for Individuals; Assessment of Intervention Implementation; and Socioemotional Considerations. These themes and their subthemes are discussed below with a focus on aspects of PRT that are viewed as positive versus negative and how PRT can be reformed to increase its social validity.

### Respect for Individuals

This theme contained three subthemes: Overemphasis on Spoken Language, Individualization of Intervention, and Interpretation of Child Behavior.

#### Overemphasis on Spoken Language

This subtheme echoes concerns brought forth by autistic advocates that, though providers may say they aim to promote communication, the ultimate goal of behavioral intervention is to actually make autistic people appear neurotypical by forcing them to speak (e.g., Neurodivergent K, 2013; Sequenzia, 2016; Wilkenfeld & McCarthy, 2020). Though PRT principles can certainly be used to teach, encourage, and reinforce any communicative act, the intervention was initially developed to promote spoken language, and most PRT studies have continued to prioritize this method of communication (Bozkus-Genc & Yucesoy-Ozkan, 2016; Ona et al., 2020). To our knowledge, no studies evaluate PRT used to teach alternative methods of communication (e.g., AAC devices, sign language, etc.). It will be prudent for clinicians to ensure that spoken language is not taught at the expense of other communication, but rather dependent on child and family preferences and child abilities. Psychoeducation regarding the importance of all modes of communication should be provided to parents. Additionally, it is important for clinicians and parents to continually evaluate the most reasonable and preferred communication modality for the child throughout the course of intervention (recognizing that this may change in different settings and across time), rather than forging onward with vocal communication at all costs.

#### Individualization of Intervention & Interpretation of Child’s Behavior

PRT’s emphasis on child choice appears to make it excel in terms of Individualization of Intervention, as many participants pointed out how sometimes the children in the videos appeared happy, but that not all autistic individuals would enjoy certain activities. Therefore, clinicians must keep in mind the necessity of tailoring interventions to the child’s sensory needs (Dynia et al., 2022)—not just in terms of activities, but also with regards to the environmental set-up (room lights, ambient sounds, etc.). This requires frequent check-ins and constant re-evaluation of the child’s sensory needs and preferences, and consultation with an occupational therapist is encouraged. Good intervention and implementation of PRT (or any intervention model) should address this through the use of preference assessments and responsiveness to the child’s needs. However, participants' emphasis on this subtheme highlights that this should be at the forefront of intervention programs. Additionally, the subtheme of Interpretation of Child’s Behavior appears related to the concept of the double empathy problem (Milton, 2012) and the difficulty non-autistic professionals might have understanding autistic children’s perspectives. This highlights the importance of considering the autistic perspective and social validity when working in a clinical or educational capacity.

### Assessment of Intervention Implementation

Study participants’ feedback zeroed in on three specific aspects of PRT fidelity: Following the Child’s Lead, Contingent Reinforcement, and Reinforcing Attempts. Two additional subthemes related to intervention implementation were also generated: Amount of Engagement and “Where’s the Intervention?”.

#### Following the Child’s Lead

With regards to following the child’s lead, some participants highlighted how adults received consent from the children they were interacting with. This focus on consent is consistent with advocates’ concerns that behavioral intervention is often done *to* a child, whether or not they find it acceptable (Sparrow, 2016; Stop ABA & Support Autistics, 2019). Including an explicit check-for-consent in PRT (and other NDBI) procedures would likely increase their social validity. However, in regard to the target of specific skills (e.g., turn taking, washing hands, brushing teeth) many young neurotypical children may not explicitly consent (and may even protest) being taught these skills, even though they are useful (especially with regard to health and safety). As such, requiring explicit consent from young autistic children for every aspect of intervention may not be feasible or appropriate. That being said, it is necessary that researchers and clinicians ensure consent is obtained whenever possible. In situations where explicit consent is not or cannot be obtained, the target goal must be generally important to autistic people and not antithetical to the autistic way of being (Schuck et al., 2021). It should also be noted that consent can be obtained in multiple ways in addition to verbal speech, such as gestures, body language, or spontaneous continuation of an activity.

On the other hand, 40% of participants noted instances where it appeared that adults were *not* following the child’s lead, particularly when activities were stopped or interrupted in order for adults to prompt the child to say something. These critiques speak to the issue of adults intruding upon children’s play in order to insert a “learning opportunity.” This is something PRT fidelity guidelines allow, though some versions of PRT have begun emphasizing the need for the adult to *enhance* the activity with a learning opportunity, as opposed to interrupting it (Vernon et al., 2019). These newer methods explicitly recommend gentler and more natural ways of gaining shared control, which may be more in line with participants' desire to avoid imposing on children.

#### Contingent Reinforcement

Contingent Reinforcement was another area where participants provided both positive/neutral and negative feedback. Reinforcement is the basis of all behavioral intervention models. However, natural reinforcement is one of the main components separating PRT (and other NDBIs) from other intervention packages (e.g., DTT). The fact that some participants noted a distaste for reinforcement in general implies a negative opinion of all behavioral intervention models, in line with other critics (e.g., Kohn, 2020; Mottron, 2017). It is important to note that even provision of the most natural reinforcement may be frowned upon, as some participants felt that access to fun activities should not be contingent upon completing a task, such as saying a word. However, other participants praised the use of natural reinforcement, suggesting this may be an aspect of PRT and NDBIs with the potential to be socially valid, at least in comparison to the use of external reinforcement. Given the disagreement regarding this component, it is important that clinicians pay attention to how much reinforcement is used (through deliberately creating opportunities for learning) and how often the child is given free access. This may be at least partially remedied by the provision of “freebies,” where children are given access to activities without imposition of demands (Vernon et al., 2019). Though reinforcement is inherent in everyday life, more thought must be given to the appropriate balance of adult-created learning opportunities and free play in interventions used for autistic children. Natural reinforcement may also be seen as more acceptable if learning opportunities themselves occur naturally and are not forced (see the above discussion on shared control).

#### Reinforcing Attempts

Participants responded positively when (non-perfect) attempts were reinforced, suggesting that it is a socially valid practice. However, participants appeared displeased when attempts were not reinforced (for example, during acquisition tasks where the adult pushed for “better” responding). Though reinforcing attempts is a hallmark of PRT, attempts are not *always* reinforced, particularly in acquisition tasks or when the child’s utterances do not naturally start to approximate the words they mean to speak. When given an acquisition task, children are pushed for a response that is a *bit* more difficult for them to produce in order to promote learning. Usually, this means pushing them to do something requiring effort but still within the child’s repertory. In the case of a non-speaking child who does not reliably approximate words and who responds to all prompts with a generalized sound, this means prompting them until they make a different utterance (Jobin & Schreibman, 2020; Koegel et al., 2003), even though they may not understand what is being asked of them. Participants found this kind of shaping to be not socially valid, suggesting that the PRT procedures for teaching first words may need to be reevaluated. One potential way to at least partially address this concern is to explicitly prime the child with what is to be expected (i.e., telling the child when you are going to push for a certain response). For instance, if a clinician previously reinforced a generalized sound, but is going to begin shaping these sounds, letting the child know by saying something like, “Let’s see if you can make a different sound now” could increase acceptability, though this needs further evaluation.

#### Amount of Engagement

Participants also commented on the engagement level of the child, suggesting that clinicians should be sensitive to this and adapt their behavior as necessary. For example, if children look bored, adults should try switching to another activity. On the other hand, adults should also be aware of whether they are being “overbearing,” and should be sure to give the child space and time to actively participate. As comments related to engagement were both positive and negative, making this a more explicit part of PRT (i.e., including it in measures of fidelity) might make PRT more socially valid for autistic individuals.

#### “Where’s the Intervention?”

Several participants remarked that they did not see any “intervention” in the videos, and the interaction simply seemed like playing. This suggests that, at least in some cases, implementation of PRT can mirror natural, positive adult–child interactions and real-life reinforcement contingencies. If one is to assume that natural adult–child play time is an acceptable form of interaction, it thus follows that PRT has the potential to be a highly socially valid intervention, since sometimes it does not even appear to be an intervention at all. Naturalistic implementation is indeed one of the aspects of NDBIs that researchers feel enhances its social validity (Gengoux et al., 2020). However, as mentioned above, adhering to PRT fidelity does not always guarantee that the interaction feels natural to the child, and natural reinforcement can still feel stilted if the entire learning opportunity itself is overly orchestrated. Clinicians should thus be attuned to how natural the interaction really feels. Furthermore, just because the PRT *procedures* shown in the videos might sometimes be considered naturalistic enough to be socially valid does not guarantee that the *goals* and *outcomes* of the intervention are also socially valid. These other aspects of social validity must be further explored (Snodgrass et al., 2018).

### Socioemotional Considerations

Three subthemes were generated with respect to the theme of socioemotional considerations: Child Affect, Parent–child Bonding, and Intervention Causes Harm.

#### Child Affect and Parent–Child Bonding

A majority of study participants commented that the children looked happy, though over a third also mentioned a child’s display of negative affect. This once again indicates that PRT has the capacity to be socially valid, but is not always seen as acceptable. Similar to discussions presented above, clinicians must be attuned to child affect, and if negative affect is shown, other activities—or even other interventions or procedures—should be introduced. This is particularly true when teaching children how to communicate; though some negative affect may accompany learning non-preferred but necessary skills (e.g., brushing teeth, looking both ways before crossing the street; again see Schuck et al., 2021 for a discussion of when it is appropriate to use NDBIs to work on non-preferred skills), it is imperative that children not associate communication with negative feelings. Clinicians must therefore constantly assess how children are feeling about interventions, for example by asking them (if appropriate), monitoring affect (e.g., Robinson, 2011), or using an alternative method (e.g., Tesfaye et al., 2019).

Similarly, parents and clinicians should be aware that simply following the PRT procedures will not automatically result in a bonding experience. The relationship between the adult and child should continually be assessed in the same way other outcome variables are measured. If it appears a positive relationship is not being fostered, changes should be implemented. Additionally, the suggestions outlined above (e.g., evaluating appropriate level of engagement, providing a better balance between learning opportunities and free play) might increase the likelihood of a positive adult–child bond.

#### Intervention Causes Harm

Importantly, a number of participants highlighted that the intervention shown in the videos could cause harm. Needless to say, an intervention cannot be socially valid if it is causing harm. However, little empirical evidence from intervention research currently exists regarding the notion that PRT and other NDBIs cause reduced self-esteem or other long-term harms, despite many autistic individuals’ insistence that ABA can be abusive. One likely reason for this is the lack of social validation of ABA interventions (including NDBIs) in autism research. That is, it is not that these interventions are necessarily harmless; it is that researchers are not asking the right questions in order to assess possible harm. To address this issue, clinicians must more consistently incorporate autistic people’s (i.e., the intervention recipient’s *and* advocates’/consultants’) perspectives when designing interventions such that intervention goals and procedures are not viewed as harmful from the beginning. Social validity should also be assessed continually throughout the intervention. Such continual evaluation will allow for potential or real harms to be quickly identified and remedied (Schwartz & Baer, 1991). Similarly, clinicians and researchers need to more systematically assess adverse events/effects during and after the course of interventions (see Bottema-Beutel et al., 2021b; Dawson & Fletcher-Watson, 2021). While adverse event reporting is not equivalent to social validity assessment [though understanding unanticipated outcomes is sometimes considered an important function of social validity (Strain et al., 2012)], we argue that any assessment of social validity is incomplete without reporting of adverse events/effects. It is evident that additional work is necessary to investigate the long-term benefits and harms of behavioral interventions, in order to fully evaluate the effects of intervention on autistic people, optimize intervention recommendations, and avoid adverse outcomes.

## Conclusion

Social validity concerns stakeholder opinions regarding the acceptability of intervention goals, procedures, and outcomes (Wolf, 1978). Our findings illuminate many areas wherein specific PRT principles (e.g., reinforcing attempts, following the child’s lead) were seen as socially valid and could potentially lead to positive *outcomes* (e.g., positive child affect, parent–child bonding). This indicates the PRT framework has the *potential* to be socially valid. However, participants also highlighted instances where they felt PRT principles were not adhered to (for example, with regards to not always following the child’s lead), even though the adults met PRT fidelity. This suggests that the nuances of the implementation of an intervention are key in social validity and that meeting fidelity is likely not enough to ensure that adults are implementing PRT in an acceptable way. Furthermore, participants were concerned that some aspects of PRT could be detrimental, both in terms of intervention *goals* (e.g., its emphasis on spoken language) and *procedures* (such as not reinforcing some attempts during acquisition tasks). Therefore, just like other models of ABA (e.g., Shepley & Grisham-Brown, 2019), PRT must continue to be reformed and its implementation must be individualized for it to be truly socially valid to the community it intends to serve. It is also important to note that the findings in this study regarding social validity of intervention goals and procedures go beyond PRT, NDBIs, and even behavioral intervention, and can also be applied more broadly across disciplines, such as speech and language therapy, occupational therapy, and preschool and elementary classrooms.

### Limitations and Future Research

Though this study helps shed light on the social validity of PRT, our findings should be considered preliminary and viewed within the context of several methodological limitations. Firstly, our overwhelmingly White, female, and later-diagnosed sample is not representative of the autistic population as a whole, particularly the subset of individuals that receive behavioral interventions such as PRT. It is highly likely that other demographic groups would have differing opinions of the intervention shown in the videos. To ensure these populations are reached, future iterations of this research should be conducted in collaboration with community organizations (as is recommended by Shaia et al., 2020) and should aim to recruit a diverse, fully representative sample of autistic individuals. Additionally, mention of “behavioral intervention” may have turned some potential participants away, given the negative views of ABA held by many in the autistic community. On the other hand, those with strong negative opinions on ABA may have actually been more likely to participate so that they could provide us with such feedback. Obtaining information regarding participants’ attitudes toward topics such as ABA, autism, and neurodiversity will be helpful for future studies in assessing how these traits influence feelings toward particular interventions.

Another limitation of the current study is the brevity of the video clips. Though we intentionally did not provide any details about the intervention or the children in the videos in order to not prime participants, the lack of context may have limited participants’ ability to give feedback, as a number of participants (n = 65) indicated that they wished they had more information. Furthermore, from a qualitative standpoint, our data was slightly limited by the fact that participants’ responses were only short free-response texts. This somewhat hindered our ability to make meaning from responses, resulting in some thinner themes that functioned more like topic domains (Braun & Clark, 2020). Future studies should thus employ more rigorous qualitative methods, such as interviews or focus groups, where richer data could help uncover the nuances of why some participants viewed the videos mostly positively, while others viewed them mostly negatively. Additionally, longer videos and/or case vignettes depicting all intervention procedures along with background information could be provided (e.g., What are the goals of the intervention? What are the intervention outcomes and how are they assessed? Did the child consent to the activity in the video? What skills are in the child’s repertoire?). This would help ensure that *all* aspects of the social validity construct are assessed (i.e., the intervention’s goals, procedures, and outcomes; Snodgrass et al., 2018; Wolf, 1978). Another way to build on our preliminary findings is to show participants randomized videos of both intervention sessions and unstructured play. This would help reduce the impact of any potential participant biases toward NDBIs and may also help us better understand the extent to which PRT and other NDBIs approximate the natural environment. Lastly, while subjective evaluation from stakeholders not involved in the intervention (such as the participants in this study) is certainly a valuable form of social validity (Kazdin & Matson, 1981), it is of course also necessary to elicit the perspectives of the intervention recipients themselves. Combining social validity data from multiple sources will help guide clinicians, teachers, and families to provide the best, most acceptable intervention to the autistic clients they serve.

## Footnotes

1 The science of applied behavior analysis (ABA) can have many applications, including treatment of substance use disorders, improving health and fitness, and running a successful business/organization (Behavior Analyst Certification Board, 2022). However, the term “ABA” is commonly associated with the use of behavioral intervention for autistic children. As such, in many professional and lay circles, “ABA” is often colloquially used to refer broadly to behavioral intervention practices used with autistic children. While we recognize that “ABA” does not refer to any one intervention, we sometimes invoke use of the colloquial “ABA” in order to be in line with the terminology often used by those in the community (for example, *Spectrum News* uses “ABA” to talk about behavioral intervention for autistic individuals (Devita-Raeburn, 2016) as does the popular hashtag #YesAllABA).
